# Association of accelerometer-measured physical activity intensity, sedentary time, and exercise time with incident Parkinson’s disease

**DOI:** 10.1038/s41746-023-00969-7

**Published:** 2023-11-28

**Authors:** Mengyi Liu, Xiaoqin Gan, Ziliang Ye, Yuanyuan Zhang, Panpan He, Chun Zhou, Sisi Yang, Yanjun Zhang, Xianhui Qin

**Affiliations:** Division of Nephrology, Nanfang Hospital, Southern Medical University; National Clinical Research Center for Kidney Disease; State Key Laboratory of Organ Failure Research; Guangdong Provincial Institute of Nephrology; Guangdong Provincial Key Laboratory of Renal Failure Research, Guangzhou, 510515 China

**Keywords:** Parkinson's disease, Risk factors

## Abstract

Evidence regarding the association between physical activity and Parkinson’s disease (PD) risk is generally limited due to the use of self-report questionnaires. We aimed to quantify the separate and combined effects of accelerometer-measured light physical activity (LPA), moderate-to-vigorous physical activity (MVPA), sedentary time and exercise timing with incident PD. 96,422 participants without prior PD and with usable accelerometer data were included from UK Biobank. Time spent in sedentary activity, LPA, MVPA, and exercise timing were estimated using machine learning models. The study outcome was incident PD. Over a median follow-up duration of 6.8 years, 313 participants developed PD. There was a L-shaped association for LPA and MVPA, and a reversed L-shaped association for sedentary time, with the risk of incident PD (all *P* for nonlinearity < 0.001). Similar trends were found across three time-windows (morning, midday-afternoon, and evening). Compared with those with both low LPA (<3.89 h/day) and low MVPA (<0.27 h/day), the adjusted HR (95% CI) of PD risk was 0.49 (0.36–0.66), 0.19 (0.36–0.66) and 0.13 (0.09–0.18), respectively, for participants with high MVPA only, high LPA only, and both high LPA and high MVPA. Moreover, participants with both low LPA and high sedentary time (≥9.41 h/day) (adjusted HR, 5.59; 95% CI: 4.10–7.61), and those with both low MVPA and high sedentary time (adjusted HR, 3.93; 95% CI: 2.82–5.49) had the highest risk of incident PD. In conclusion, regardless of exercise timing (morning, midday-afternoon, and evening), there was an inverse association for accelerometer-measured MVPA and LPA, and a positive association for sedentary time, with incident PD.

## Introduction

Parkinson’s disease (PD) is the second most common neurodegenerative disease (after Alzheimer’s disease) characterized by the progressive loss of dopaminergic neurons in the substantia nigra and in other neuronal systems^1,2^. PD affects 2 to 3% of the global population aged over 65, and the incidence of PD continues to rise in almost every part of the world with the increase of aging population^3,4^. In the absence of effective drugs to halt or retard neurodegeneration, developing non-pharmacological strategies (i.e., lifestyle approaches) to prevent the onset of PD is essential.

Physical activity (PA), defined as any bodily movement produced by skeletal muscles resulting in energy expenditure^5^, has long been known to reduce mortality and lower the risk of other neurodegenerative diseases such as Alzheimer’s disease and cognitive decline^6–9^. Although intensive PA has been reported to be associated with neuroprotective and neurorestorative effects in the nigrostriatal dopaminergic system^10^, previous cohort studies designed to examine the relationship between PA and the risk of PD have yielded inconsistent results. A recent meta-analysis of previous prospective studies showed that there was an inverse dose-response association of total PA and moderate-to-vigorous PA (MVPA) with PD risk in men, but not in women^11^, while a subsequent study reported that higher total PA was significantly associated with a lower PD incidence in women^12^. Of note, all of these studies used self-reported measures of PA, which are more prone to recall bias and, therefore, a weak surrogate to measure activity and capture the true magnitude and nature of the association between PA and PD risk. Moreover, due to the inability of self-reported questionnaires to accurately measure light intensity PA (LPA)^13,14^, there is limited evidence for the association between LPA and PD risk, despite LPA being the predominant form of activity in daily life and a more feasible behavioral goal for middle-aged and older adults^15^.

In addition, there is growing evidence that almost all physiological and biochemical processes in the human body follow circadian rhythms^16^, so there is a need to further clarify whether the health benefits of PA depend on the temporal distribution of PA throughout the day. While previous studies have suggested that PA at specific times may be beneficial in lowering blood glucose levels and activating PA metabolic pathways^17,18^, there is no consistent evidence that equivalent amounts of PA in the morning, midday-afternoon, and evening may have different effects on health outcomes^19^. More importantly, few studies have assessed the relationship between PA at different times of the day (morning, midday-afternoon, and evening) and PD risk.

Moreover, sedentary time is another major part of daily activities for the general population^15^ and is thought to impair glucose and lipid metabolism^20^, both of which are risk factors for PD^21^. Nevertheless, there is little direct evidence of the association between sedentary time and PD risk. Furthermore, given that different combinations of PA and sedentary time are reasonable during waking times, and may potentially lead to a gradient of health consequences^22,23^, it is important to assess the role of the interrelationship between PA and sedentary time on PD risk.

Overall, due to the recall bias of traditional self-reported PA measurements and limitations such as the inability to accurately collect LPA, exercise time, and sedentary time, using new digital and mobile technologies to accurately address the above gaps in knowledge becomes a necessary option. Of note, embedded sensors in wearable technology can capture data remotely, passively, and continuously, and various algorithms may then be applied to the acceleration signal to more accurately estimate LPA, MVPA, exercise time and sedentary time. However, no studies have fully evaluated the relationship between these various objective accelerometer-generated PA information and the risk of incident PD.

As such, using data from the UK Biobank, we aimed to examine the individual and combined effects of accelerometer-measured LPA, MVPA, sedentary time and exercise timing on the risk of incident PD.

## Results

### Study participants and population characteristics

Of the 96,422 participants included (Supplementary Fig. 1), the mean age was 56.1 (SD, 7.8) years, and 54,323 (56.3%) were female. The mean times spent in LPA, MVPA, and sedentary activity were 5.1 (SD, 1.6), 0.7 (SD, 0.6), and 9.4 (SD, 1.8) hours/day, respectively.

During a median follow-up of 6.8 years (interquartile range, 6.2–7.3 years), 313 (0.3%) participants developed incident PD, of which 98.4% were ascertained by hospital admissions recodes. As shown in Table 1, compared with participants without incident PD, those with incident PD were older and more likely to be male, tended to have lower income, and had a higher prevalence of hypertension, diabetes and CVD.

**Table 1:** General characteristics of study participants by incidence of Parkinson’s disease^a^.

Characteristics	All participants	Non-cases	Incident CKD cases	*P* value
*N*	96422	96109	313	
Age, years	56.1 (7.8)	56.1 (7.8)	62.9 (4.9)	<0.001
Male, *n* (%)	42099 (43.7)	41885 (43.6)	214 (68.4)	<0.001
White, *n* (%)	93121 (96.6)	92814 (96.6)	307 (98.1)	0.312
Recruitment centers, *n* (%)				<0.001
England	86581 (89.8)	86278 (89.8)	303 (96.8)	
Scotland	6242 (6.5)	6233 (6.5)	9 (2.9)	
Wales	3599 (3.7)	3598 (3.7)	1 (0.3)	
Townsend deprivation index	−1.7 (2.8)	−1.7 (2.8)	−2 (2.7)	0.060
Employed, *n* (%)	90906 (94.3)	90602 (94.3)	304 (97.1)	0.074
Income (<£31,000), *n* (%)	33662 (34.9)	33528 (34.9)	134 (42.8)	0.009
Education (college or university), *n* (%)	41445 (43.0)	41316 (43.0)	129 (41.2)	0.089
Smoking status, *n* (%)				0.075
Current	54920 (57.1)	54742 (57.1)	178 (57.1)	
Previous	34580 (36.0)	34458 (35.9)	122 (39.1)	
Never	6674 (6.9)	6662 (6.9)	12 (3.8)	
Alcohol drinking, *n* (%)				0.422
>4 times/week	22031 (22.9)	21947 (22.9)	84 (26.8)	
3–4 times/week	25058 (26.0)	24988 (26.0)	70 (22.4)	
1–2 times/week	24181 (25.1)	24105 (25.1)	76 (24.3)	
<1 time/week	19621 (20.4)	19556 (20.4)	65 (20.8)	
Never	5460 (5.7)	5442 (5.7)	18 (5.8)	
Body mass index, kg/m2	26.7 (4.5)	26.7 (4.5)	26.7 (4.2)	0.991
Pre-existing chronic conditions, *n* (%)				
Hypertension	26301 (27.5)	26176 (27.5)	125 (40.2)	<0.001
Diabetes	4041 (4.2)	4013 (4.2)	28 (8.9)	<0.001
Cardiovascular disease	6745 (7.0)	6693 (7.0)	52 (16.7)	<0.001
Season of accelerometer wear, *n* (%)				0.572
Spring	22038 (22.9)	21959 (22.8)	79 (25.2)	
Summer	25631 (26.6)	25544 (26.6)	87 (27.8)	
Autumn	28503 (29.6)	28414 (29.6)	89 (28.4)	
Winter	20250 (21.0)	20192 (21.0)	58 (18.5)	
Light physical activity, hours/day	5.1 (1.6)	5.1 (1.6)	3.7 (1.8)	<0.001
Moderate-to-vigorous physical activity, hours/day	0.7 (0.6)	0.7 (0.6)	0.6 (0.6)	<0.001
Sedentary time, hours/day	9.4 (1.8)	9.4 (1.8)	10.5 (2.3)	<0.001

^a^Data are expressed as mean (SD), or *n* (%), accordingly.

### Association of LPA, MVPA and sedentary time with incident PD

**Fig. 1:**
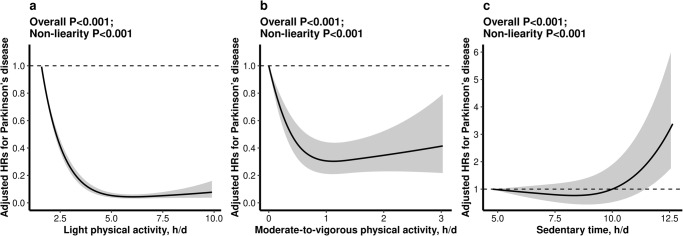
The dose-response association of light and moderate-to-vigorous physical activity and sedentary time with the risk of incident Parkinson’s disease. There was a nonlinear association of light (**a**) and moderate-to-vigorous physical activity (**b**) and sedentary time (**c**) with the risk of incident Parkinson’s disease. HR indicates hazard ratio. In each plot, the solid line indicates how Parkinson’s disease incidence varies as a function of time spent in physical activity or sedentary behavior, while the dashed lines are confidence intervals. All results were adjusted for age, sex, ethnicities, recruitment center, Townsend Deprivation Index, educational attainment, household income, employment, smoking status, alcohol consumption, and body mass index, pre-existing chronic conditions (hypertension, diabetes and cardiovascular disease), and the season of accelerometer wear.

As shown in Fig. 1, RCS Cox regression showed that there was a L-shaped association for LPA and MVPA, and a reversed L-shaped association for sedentary time, with the risk of incident PD (all *P* for nonlinearity < 0.001).

Consistently, when PA and sedentary time were assessed as quartiles, compared with participants in the first quartile, those in the second (3.89- < 4.93 h/day; adjusted HR, 0.29; 95%CI: 0.22, 0.39), third (4.93- < 6.08 h/day; adjusted HR, 0.19; 95%CI: 0.13, 0.27), and fourth (≥6.08 h/day; adjusted HR, 0.18; 95% CI: 0.12, 0.27) quartiles of LPA, and those in the second (0.27- < 0.55 h/day; adjusted HR, 0.56; 95%CI: 0.42, 0.75), third (0.55- < 0.96 h/day; adjusted HR, 0.49; 95%CI: 0.36, 0.68), and fourth (≥0.96 h/day; adjusted HR, 0.38; 95%CI: 0.27, 0.53) quartiles of MVPA, had a significantly lower risk of incident PD, while those in the third (9.41- < 10.61 h/day; adjusted HR, 1.45; 95%CI: 1.00, 2.11) and fourth (≥10.61 h/day; adjusted HR, 2.91; 95%CI: 2.06, 4.10) quartiles of sedentary time had a significantly higher risk of incident PD (Table 2).

**Table 2:** Association of light and moderate-to-vigorous physical activity and sedentary time with the risk of incident Parkinson’s disease.

Exposure, hours/day	Total	Events (rate^a^)	Crude Model		Adjusted Model^b^	
			HR (95%CI)	*P* value	HR (95%CI)	*P* value
Light physical activity						
Quartiles						
Q1 (<3.89)	24097	193 (12.2)	ref		ref	
Q2 (3.89-to<4.93)	24111	55 (3.4)	0.28 (0.21, 0.38)	<0.001	0.29 (0.22, 0.39)	<0.001
Q3 (4.93-to<6.08)	24100	35 (2.2)	0.18 (0.13, 0.26)	<0.001	0.19 (0.13, 0.27)	<0.001
Q4 (≥6.08)	24114	30 (1.9)	0.15 (0.10, 0.22)	<0.001	0.18 (0.12, 0.27)	<0.001
*P* for trend			<0.001		<0.001	
Moderate-to-vigorous physical activity						
Quartiles						
Q1 (<0.27)	24089	119 (7.6)	ref		ref	
Q2 (0.27-to<0.55)	24117	72 (4.5)	0.59 (0.44, 0.79)	<0.001	0.56 (0.42, 0.75)	<0.001
Q3 (0.55-to<0.96)	24088	66 (4.1)	0.54 (0.40, 0.73)	<0.001	0.49 (0.36, 0.68)	<0.001
Q4 (≥0.96)	24128	56 (3.5)	0.46 (0.33, 0.63)	<0.001	0.38 (0.27, 0.53)	<0.001
* P* for trend			<0.001		<0.001	
Sedentary time						
Quartiles						
Q1 (<8.19)	24106	46 (2.9)	ref		ref	
Q2 (8.19-to<9.41)	24089	45 (2.8)	0.98 (0.65, 1.48)	0.930	0.88 (0.58, 1.33)	0.550
Q3 (9.41-to<10.61)	24121	74 (4.6)	1.62 (1.12, 2.34)	0.011	1.45 (1.00, 2.11)	0.049
Q4 (≥10.61)	24106	148 (9.3)	3.27 (2.35, 4.55)	<0.001	2.91 (2.06, 4.10)	<0.001
*P* for trend			<0.001		<0.001	

^a^Incidence rates per 10000 person-years.

^b^Adjusted for age, sex, ethnicities, recruitment center, Townsend Deprivation Index, educational attainment, household income, employment, smoking status, alcohol consumption, and body mass index, pre-existing chronic conditions (hypertension, diabetes and cardiovascular disease), and the season of accelerometer wear.

The results did not change substantially by further excluding participants within 2 years of follow-up, or accounting for the competing risk of death (Supplementary Table 1). Moreover, we observed a U-shaped association for non-occupational computer use and a positive association for TV-watching with incident PD (Supplementary Fig. 2).

### Combined effect of LPA and MVPA on the risk of incident PD

Based on the incidence of PD and the HRs in Table 2, the quartiles of PA and sedentary time were further combined to generate a binary variable. As expected, low PA (LPA < 3.89 h/day, and MVPA < 0.27 h/day) and high sedentary time (≥9.41 h/day) were significantly associated with a higher risk of incident PD (Supplementary Table 2).

When both LPA and MVPA were considered, compared with those with both low LPA and low MVPA, the adjusted HR (95%CI) of PD risk was 0.49 (0.36,0.66), 0.19 (0.36, 0.66) and 0.13 (0.09, 0.18), respectively, for participants with high MVPA only, high LPA only, and with both high LPA and high MVPA (Table 3). There was an additive interaction (*P* values for RERI < 0.001), but not multiplicative interaction (*P* for interaction = 0.168) between LPA and MVPA on PD risk (Table 3).

**Table 3:** Combined effect of light and moderate-to-vigorous physical activity (Low *vs*. High levels) on the risk of incident Parkinson’s disease^a^.

Light physical activity	Moderate-to-vigorous physical activity	Total	Events (rate^b^)	Adjusted Model^c^		Additive interaction (RERI)^c^		Multiplicative interaction^c^	
				HR (95%CI)	*P* value	Estimates (95%CI)	*P* value	Estimates (95%CI)	*P* value
Combination of light and moderate-to-vigorous physical activity						0.46 (0.29, 0.62)	<0.001	1.41 (0.86, 2.30)	0.168
Low	Low	7267	85 (18.2)	ref					
Low	High	16830	108 (9.6)	0.49 (0.36, 0.66)	<0.001				
High	Low	16822	34 (3.1)	0.19 (0.12, 0.28)	<0.001				
High	High	55503	86 (2.3)	0.13 (0.09, 0.18)	<0.001				

^a^Low physical activity: <3.89 h/day (Quartile 1) for Light physical activity, and <0.27 h/day (Quartile 1) for Moderate-to-vigorous physical activity.

^b^Incidence rates per 10000 person-years.

^c^Adjusted for age, sex, ethnicities, recruitment center, Townsend Deprivation Index, educational attainment, household income, employment, smoking status, alcohol consumption, and body mass index, pre-existing chronic conditions (hypertension, diabetes and cardiovascular disease), and the season of accelerometer wear.

### Combined effect of PA (LPA and MVPA) and sedentary time on the risk of incident PD

When both PA (LPA and MVPA) and sedentary time were considered, compared with those with high LPA and low sedentary time, participants with both low LPA and high sedentary time had the highest risk of incident PD (adjusted HR, 5.59; 95% CI: 4.10, 7.61; Table 4). Similarly, comparted to participants with high MVPA and low sedentary time, the highest risk of incident PD was found in those with both low MVPA and high sedentary time (adjusted HR, 3.93; 95%CI: 2.82, 5.49) (Table 4). However, there were no significant interaction between PA and sedentary time on either additive (all *P* values for RERI > 0.05) or multiplicative scales (All *P* for interaction > 0.05; Table 4).

**Table 4:** Combined effect of light and moderate-to-vigorous physical activity (Low *vs*. High levels) and sedentary time (Low vs. High levels) on the risk of incident Parkinson’s disease^a^.

Physical activity	Sedentary time	Total	Events (rate^b^)	Adjusted Model^c^		Additive interaction (RERI)^c^		Multiplicative interaction^c^	
				HR (95%CI)	*P* value	Estimates (95% CI)	*P* value	Estimates (95% CI)	*P* value
Combination of light physical activity and sedentary time						0.07 (−1.97, 2.11)	0.947	1.37 (0.81, 2.34)	0.236
High	Low	44584	61 (2.1)	ref					
High	High	27741	59 (3.2)	1.55 (1.08, 2.22)	0.017				
Low	Low	3611	30 (12.6)	4.97 (3.18, 7.76)	<0.001				
Low	High	20486	163 (12.1)	5.59 (4.10, 7.61)	<0.001				
Combination of moderate-to-vigorous physical activity and sedentary time						0.71 (−0.49, 1.91)	0.245	1.13 (0.67, 1.91)	0.641
High	Low	38800	64 (2.5)	ref					
High	High	33533	130 (5.8)	2.19 (1.62, 2.97)	<0.001				
Low	Low	9395	27 (4.4)	2.03 (1.29, 3.22)	0.002				
Low	High	14694	92 (9.6)	3.93 (2.82, 5.49)	<0.001				

^a^Low physical activity: <3.89 h/day (Quartile 1) for Light physical activity, and <0.27 h/day (Quartile 1) for Moderate-to-vigorous physical activity; Low sedentary time: <9.41 h/day (median).

^b^Incidence rates per 10000 person-years.

^c^Adjusted for age, sex, ethnicities, recruitment center, Townsend Deprivation Index, educational attainment, household income, employment, smoking status, alcohol consumption, and body mass index, pre-existing chronic conditions (hypertension, diabetes and cardiovascular disease), and the season of accelerometer wear.

### Association of LPA, MVPA and sedentary time with incident PD among different time-windows (morning, midday-afternoon, and evening)

**Fig. 2:**
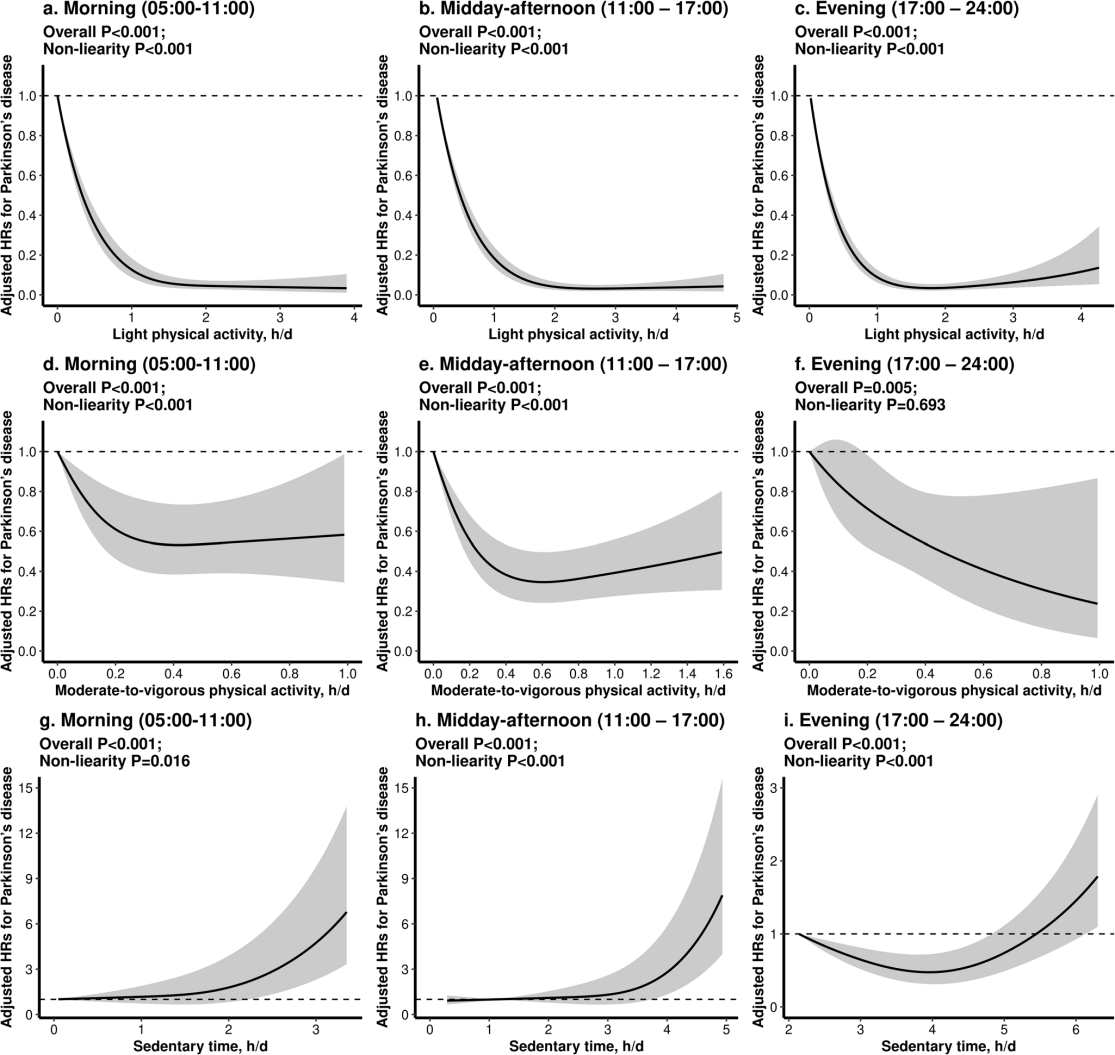
The dose-response associations between light and moderate-to-vigorous physical activity and sedentary time within three time-windows and the risk of incident Parkinson’s disease. There were dose-response associations between light (**a**–**c**) and moderate-to-vigorous physical activity (**d**–**f**) and sedentary time (**g**–**i**) within three time-windows (morning, midday-afternoon, and evening) and the risk of incident Parkinson’s disease. HR indicates hazard ratio. In each plot, the solid line indicates how Parkinson’s disease incidence varies as a function of time spent in physical activity or sedentary behavior, while the dashed lines are confidence intervals. All results were adjusted for age, sex, ethnicities, recruitment center, Townsend Deprivation Index, educational attainment, household income, employment, smoking status, alcohol consumption, and body mass index, pre-existing chronic conditions (hypertension, diabetes and cardiovascular disease), and the season of accelerometer wear.

As shown in Fig. 2, there was a similar L-shaped association between LPA and incident PD in the three time-windows (morning, midday-afternoon, and evening). There was a similar L-shaped association between MVPA and incident PD in the morning and midday-afternoon period, while there was a linear inverse association between MVPA and incident PD in the evening period. There was a similar positive association between sedentary time and incident PD in the morning and midday-afternoon period, while a J-shaped association in the evening period.

### Stratified analyses

Stratified analyses were performed to further assess the relationship of PA and sedentary time with the risk of PD in various subgroups (Supplementary Table 3). None of the variables, including age, sex, BMI, smoking status, alcohol drinking, and TDI, showed significant effect modifications on the association of PA or sedentary time with the risk of PD.

## Discussion

Using a large prospective cohort study, we provided evidence that regardless of the exercise timing (morning, midday-afternoon, and evening), there was an inverse association for MVPA and LPA, and a positive association for sedentary time, with the risk of PD. More importantly, participants with both high LPA (≥3.89 h/day) and high MVPA (≥0.27 h/day) had the lowest risk of PD, while participants with both low PA (LPA < 3.89 h/day and MVPA < 0.27 h/day) and high sedentary time (≥9.41 h/day) had the highest risk of PD.

A recent meta-analysis of previous prospective studies^24–29^ found an inverse dose-response association of total PA and MVPA with PD risk in men, but not in women^11^. Subsequently, the E3N Cohort Study observed a significant inverse association between total PA and PD in women^12^. In contrast, the few previous studies^25,26,28^ did not observe a significant association between LPA and PD risk. It is important to note that all of the above studies have common limitations in that they used self-reported questionnaires to assess PA, which are more prone to recall bias and have lower validity and accuracy. What’s more, the ubiquitous presence and sporadic nature of LPA makes it impossible to accurately assess through recall by self-reported questionnaires^30^. Therefore, to date, the relationship of LPA and MVPA and PD risk remains unknown. At the same time, few studies have examined the association between sedentary time and PD risk.

Based on accelerometer-derived PA, sedentary time and exercise timing (morning, midday-afternoon, and evening), our study provides some unique contributions. First, we found that objectively quantified PA, regardless of LPA or MVPA, was associated with a significantly reduced risk of PD. These findings are consistent with evidence from animal and human data that physical exercise has neuroprotective effects by promoting the expression of neuroprotective growth factors, increasing the release of neurotrophic factors, reducing damage to dopaminergic neurons in motor circuits, and reducing cellular inflammation and oxidative stress^10,31^. Moreover, we further observed an additive interaction between LPA and MVPA, suggesting that high LPA and MVPA synergistically decrease the risk of PD, and therefore, promotions targeting both LPA and MVPA may be more effective in preventing PD. Our results also suggest that participation in LPA may have a greater benefit relative to participation in MVPA. In addition, in the 1st quartile population, only a modest increase in either LPA or MVPA to 3.89 h or 0.27 h daily was associated with a substantially lower risk of PD. Since such an increase in activity is achievable in humans, our study emphasizes the potential benefits of a modest increase in MVPA to reduce the risk of PD, and further provides an alternative strategy of increasing LPA to reduce the risk of PD, as LPA is more feasible and reasonable for inactive individuals or middle-aged and elderly people. Further work is needed to confirm our results and determine causality.

Second, given that most adults spend most of their waking time inactive, the effect of sedentary time on health is gaining widespread attention. To our knowledge, no other studies have examined the association of sedentary time with PD risk. We found a nonlinear positive association between sedentary time and PD risk, with a threshold of approximately 9.4 h per day, which is similar to the threshold, 9 h per day, between daily sedentary time and all-cause mortality risk, beyond which the risk of all-cause death increases^32^. Although PA has been reported to eliminate the increased risk of death associated with high sedentary time^33^, and our study also showed that the combination of low PA and high sedentary time led to the highest risk of incident PD, we found that PA and sedentary time had neither a significant additive nor a significant multiplicative interaction effect on PD risk. It suggests that PA and sedentary time are two key and independent behavioral pathways that modify the risk of incident PD.

Third, although a growing recognition of the importance of exercise timing on health^19^, little is known about the effect of timing of exercise on PD risk. Our study showed that regardless of exercise timing (morning, midday-afternoon, and evening), higher MVPA and LPA levels were associated with a lower risk of PD, while high sedentary time was associated with a higher risk of PD. Of note, we observed a slightly lower PD risk associated with moderate sedentary time in the evening period. One possible explanation is the heterogeneity in the types of sedentary behavior, as specific components of sedentary behavior may have different effects on cognition. The mentally active types of sedentary behavior (such as reading and computer using) immediately affects cerebral hemodynamics, leading to higher blood flow velocities and higher cerebral metabolism^34,35^, and thereby contributing to improved cerebrovascular health. Consistently, in our sensitivity analysis, there was a U-shaped association between non-occupational computer using with the risk of incident PD.

Overall, the contribution of our study in terms of digital medicine is as follows: Based on the objectively measured time of different movement behavior in a free-living setting, which could be better interpretable and easier to be translated into recommendations, our study explores the individual and combined effects of accelerometer-measured LPA and MVPA and sedentary time on the risk of incident PD, suggesting that the digitization of wearable sensors may have practical value in the primary prevention of PD. Moreover, we introduced a timing phenotype of activity and found that increasing PA and decreasing sedentary time in any time windows (morning, midday-afternoon, and evening) was associated with a lower risks of PD. Our findings suggest that clinicians and healthcare systems may consider using accelerometer-measured PA and sedentary time to guide and improve strategy development and practice for prevention of PD.

This study has some limitations. Firstly, due to the nature of observational studies, despite comprehensive adjustments for a range of covariates, residual confounding cannot be completely ruled out and causality cannot be determined. Secondly, although 7-day monitoring periods have been routinely used in previous studies because they provide an opportunity to sample PA and sedentary time on both weekdays and weekend days, and achieve a greater than 80% intra-class correlations in most populations^36^, the single time-point limits any potential inferences related to within-person changes or variability in PA and sedentary time over time. Thirdly, the participants in our study were predominantly of European descent and healthier than the UK general population^37^, which may limit the generalizability of the findings to other populations. Nevertheless, a representative population is not required to validly assess the relationship between exposure and disease^37^. Due to these limitations, further studies to confirm our findings are necessary.

In conclusion, we found that regardless of exercise timing (morning, midday-afternoon, and evening), higher accelerometer-measured MVPA (≥0.27 h/day) and LPA (≥3.89 h/day) were associated with a lower risk of PD, while higher sedentary time (≥9.41 h/day) was associated with a higher risk of PD. Moreover, participants with both high LPA and high MVPA had the lowest risk of PD, and participation in LPA may have a greater benefit relative to participation in MVPA. If further confirmed, our study suggested that increasing participation in PA, regardless of intensity and exercise timing, and controlling sedentary time should be prioritized throughout one’s lifetime to prevent PD incidence. Clinicians and healthcare systems may consider the use of accelerometer-measured PA and sedentary time as a metric of health evaluation and promotion.

## Methods

### Study design and participants

As previously described^38^, the UK Biobank is a large, observational, population-based cohort recruiting half a million adult residents, aged 37–73 years, from 1 of 22 assessment centers across the United Kingdom (England, Wales, and Scotland) between 2006 and 2010. At baseline, participants were asked to complete a comprehensive questionnaire assessing sociodemographic, lifestyle, and health related information, receive physical examinations and provide biological samples. The UK Biobank was approved by the North West Research Ethics Committee (11/NW/0382) and all participants signed an informed consent.

The current analysis was restricted to a sub-sample of 103,661 participants responded to an email for the accelerometer sub-study between 2013 and 2015. Participants were excluded if the device failed to calibrate, if more than 1% of readings were ‘clipped’ which occur when the sensor’s dynamic range of ±8 g is exceeded before or after calibration, if the wear-time was insufficient (defined as the unavailability of at least 72 h of data or did not have data in each 1-hour period of the 24-hour cycle), if the average acceleration was implausibly high (>100 milli-gravity), or if the file failed to process^39,40^. Additionally, we excluded participants who had been diagnosed with dementia or PD before the end of their accelerometer wear, resulting in 96,422 participants for the final analysis (Supplementary Fig. 1).

### Exposure assessment

Between February 2013 and December 2015, participants who provided a valid email address to UK Biobank were invited at random to wear a wrist-worn accelerometer (Axivity AX3). The response rate was 45%, but participants who received accelerometry measurements showed similar baseline demographic and health-related characteristics as those who declined the measurements^40^. Participants were instructed to wear the device on their dominant wrist continuously for 1 week while continuing with their usual activities. The accelerometer captures triaxial acceleration data over 7 days at 100 Hz with a dynamic range of ±8 gravity. Time spent in sedentary activity (waking behavior at ≤1.5 metabolic equivalent of task [METs], such as driving or watching television), LPA (waking behavior at <3 METs not meeting the sedentary behavior definition, such as cooking or self-care), and MVPA (waking behavior at ≥3 METs, such as walking the dog or jogging) were estimated using machine learning models that were trained using wearable cameras and time-use diaries among 152 individuals in free-living conditions^39^.

### Outcome assessment

The study outcome was incident PD. Ascertainment of PD was based on self-reported data, diagnosis in the hospital admissions data, or cause of death recorded in the national death register, whichever was the earliest. The UK Biobank study conducted a validation study on the accuracy of code sources for identification of PD cases, which achieved a positive predictive value (PPV) of 0.91 (https://biobank.ndph.ox.ac.uk/ukb/ukb/docs/alg_outcome_pdp.pdf). The follow-up for each participant was calculated from the final date of accelerometer wear until the first date of PD diagnosis, date of death, date of loss to follow-up, or the end of follow-up, whichever came first.

### Covariates assessment

Detailed information on covariates was available through standardized questionnaires at baseline, including age, sex, ethnicities, Townsend Deprivation Index (TDI), income, education levels, employment, smoking, and alcohol drinking. Body mass index (BMI) was also measured and calculated as weight (in kilograms (kg)) divided by the square of height (in meters (m)). Pre-existing hypertension, diabetes, and cardiovascular disease (CVD) were diagnosed by baseline self-reported medical history or health records taken before the time of accelerometer measurement.

### Statistical analysis

Population characteristics, presented as mean (SD) for continuous variables or proportions for categorical variables, according to incident PD status, were compared using t-tests for continuous variables and chi-square tests for categorical variables.

Restricted cubic spline (RCS) Cox regression was performed to test for linearity and explore the shape of the dose-response relationship of PA and sedentary time with incident PD. Cox proportional hazards models were used to estimate hazard ratio (HR) and 95% confidence interval (CI) for incident PD according to the quartiles of exposures (LPA, MVPA and sedentary time) throughout the day and at different time-windows (morning, midday-afternoon, and evening), without and with adjustments for demographics (age, sex, ethnicities, recruitment center, TDI, educational attainment, household income, employment), anthropometric and lifestyle factors (smoking status, alcohol consumption, and BMI), pre-existing chronic conditions (hypertension, diabetes, and CVD), and the season of accelerometer wear. The proportional hazards assumption was checked using the Schoenfeld residuals, and no violation was found.

To test the robustness of our findings, several sensitivity analyses were also performed. First, participants within 2 years of follow-up were excluded. Second, the Fine-Gray competing risk model was used to examine the association while accounting for death as a competing risk. Third, we further explore the association between sedentary behaviors (TV-watching and non-occupational computer use) and incident PD. At baseline, the durations of sedentary behaviors were collected using a touchscreen questionnaire by asking “In a typical day, how many hours do you spend watching TV/using the computer (do not include using a computer at work)”.

In addition, the adjusted Cox proportional hazards model was used to examine the combined effect of LPA and MVPA (categorized to four groups), and PA (LPA and MVPA) and sedentary time (categorized to four groups, respectively) on PD risk. The relative excess risk due to interaction (RERI) was calculated to evaluate the interaction between PA and sedentary time on an additive scale, where an RERI equal 0 means no additive interaction and > 0 means a positive interaction^41^. Interactions on a multiplicative scale were also tested by adding a cross-product term between PA and sedentary time to the multivariable Cox models.

As additional exploratory analyses, RCS Cox regression was used to determine the dose-response relationship of PA and sedentary time within three time-windows (morning [05:00–11:00], midday-afternoon [11:00–17:00], and evening [17:00–24:00]) with incident PD. Furthermore, possible modifications of the association of PA and sedentary time with incident PD were also assessed for the following variables: age (<60 or ≥60 years), sex (females or males), BMI (<25 or ≥25 kg/m^2^), smoking status (never or ever), alcohol drinking (<1 or ≥ 1 times/week), and TDI (<median or ≥ median).

A two-tailed *P* < 0.05 was considered to be statistically significant in all analyses. Analyses were performed using R 4.1.1 software (http://www.R-project.org/).

### Reporting summary

Further information on research design is available in the Nature Research Reporting Summary linked to this article.

### Supplementary information

Reporting Summary

Supplementary Information

## Data Availability

The UK Biobank data are available on application to the UK Biobank.

## Code availability

The analytic methods, code, and study materials that support the findings of this study will be available from the corresponding authors on request.
