# Supplementary material for: Association of accelerometer-measured physical activity intensity, sedentary time, and exercise time with incident Parkinson’s disease
Source: NPJ Digit Med. 2023 Nov 28;6:224. doi: 10.1038/s41746-023-00969-7 (PMC10684568; doi:10.1038/s41746-023-00969-7)
Supplement: Supplementary file 1 — Reporting Summary [file 41746_2023_969_MOESM1_ESM.pdf]

nature portfolio

Corresponding author(s): Xianhui Qin

Last updated by author(s): Nov 8, 2023

Reporting Summary

Nature Portfolio wishes to improve the reproducibility of the work that we publish. This form provides structure for consistency and transparency in reporting. For further information on Nature Portfolio policies, see our Editorial Policies and the Editorial Policy Checklist.

Statistics

For all statistical analyses, confirm that the following items are present in the figure legend, table legend, main text, or Methods section.

n/a	Confirmed	
☐	☒	The exact sample size (*n*) for each experimental group/condition, given as a discrete number and unit of measurement
☐	☒	A statement on whether measurements were taken from distinct samples or whether the same sample was measured repeatedly
☐	☒	The statistical test(s) used AND whether they are one- or two-sided<br>*Only common tests should be described solely by name; describe more complex techniques in the Methods section.*
☐	☒	A description of all covariates tested
☐	☒	A description of any assumptions or corrections, such as tests of normality and adjustment for multiple comparisons
☐	☒	A full description of the statistical parameters including central tendency (e.g. means) or other basic estimates (e.g. regression coefficient) AND variation (e.g. standard deviation) or associated estimates of uncertainty (e.g. confidence intervals)
☐	☒	For null hypothesis testing, the test statistic (e.g. *F*, *t*, *r*) with confidence intervals, effect sizes, degrees of freedom and *P* value noted<br>*Give P values as exact values whenever suitable.*
☒	☐	For Bayesian analysis, information on the choice of priors and Markov chain Monte Carlo settings
☒	☐	For hierarchical and complex designs, identification of the appropriate level for tests and full reporting of outcomes
☐	☒	Estimates of effect sizes (e.g. Cohen's *d*, Pearson's *r*), indicating how they were calculated

Our web collection on statistics for biologists contains articles on many of the points above.

Software and code

Policy information about availability of computer code

Data collection	UK Biobank, a prospective cohort study whose data are publicly available for research use by application (https://wwwukbiobank.acuk/enable-your-research/register).
Data analysis	Analyses were performed using R 4.1.1 software (http://www.R-project.org/).

For manuscripts utilizing custom algorithms or software that are central to the research but not yet described in published literature, software must be made available to editors and reviewers. We strongly encourage code deposition in a community repository (e.g. GitHub). See the Nature Portfolio guidelines for submitting code & software for further information.

Data

Policy information about availability of data

All manuscripts must include a data availability statement. This statement should provide the following information, where applicable:

- Accession codes, unique identifiers, or web links for publicly available datasets
- A description of any restrictions on data availability
- For clinical datasets or third party data, please ensure that the statement adheres to our policy

The UK Biobank data are available on application to the UK Biobank.

Research involving human participants, their data, or biological material

Policy information about studies with human participants or human data. See also policy information about sex, gender (identity/presentation), and sexual orientation and race, ethnicity and racism.

Reporting on sex and gender	The sex information is acquired from central registry at recruitment, but in some cases updated by the participant. Accelerometer-measured physical activity and sedentary time were associated with the risk of PD in general population. We also conducted subgroup analyses on sex and found that the findings apply to males and females.
Reporting on race, ethnicity, or other socially relevant groupings	The ethnicities information is acquired from central registry at recruitment through standardized questionnaires. Ethnicities were adjusted for in the multivariable model.
Population characteristics	Of the 96,422 participants included, the mean age was 56.1 (SD, 7.8) years, and 54,323 (56.3%) were female. The mean times spent in LPA, MVPA, and sedentary activity were 5.1 (SD, 1.6), 0.7 (SD, 0.6), and 9.4 (SD, 1.8) hours/day, respectively. During a median follow-up of 6.8 years (interquartile range, 6.2-7.3 years), 313 (0.3%) participants developed incident PD, of which 98.4% were ascertained by hospital admissions recodes. Compared with participants without incident PD, those with incident PD were older and more likely to be male, tended to have lower income, and had a higher prevalence of hypertension, diabetes and CVD.
Recruitment	The UK Biobank is a population-based prospective study with over 500 000 participants aged 40-73 years recruited in 2006-2010. Participants underwent detalled baseline assessments including various socio-demographics, lifestyles, health.and physica assessments through touch-screen questionnaires and physical measurements.Further details of the study areavailable online (www.ukbiobank.ac.uk). Between February 2013 and December 2015, participants who provided a valid email address to UK Biobank were invited at random to wear a wrist-worn accelerometer (Axivity AX3). The response rate was 45%. Finally, 103 661 raw accelerometer datasets were received for data analysis.
Ethics oversight	The UK Biobank was approved by the North West Research Ethics Committee (11/NW/0382) and all participants signed an informed consent.

Note that full information on the approval of the study protocol must also be provided in the manuscript.

Field-specific reporting

Please select the one below that is the best fit for your research. If you are not sure, read the appropriate sections before making your selection.

☒ Life sciences ☐ Behavioural & social sciences ☐ Ecological, evolutionary & environmental sciences

For a reference copy of the document with all sections, see nature.com/documents/nr-reporting-summary-flat.pdf

Life sciences study design

All studies must disclose on these points even when the disclosure is negative.

Sample size	96,422 participants
Data exclusions	Participants were excluded if the device failed to calibrate, if more than 1% of readings were 'clipped' which occur when the sensor's dynamic range of ±8 g is exceeded before or after calibration, if the wear-time was insufficient (defined as the unavailability of at least 72 hours of data or did not have data in each 1-hour period of the 24-hour cycle), if the average acceleration was implausibly high (>100 milli-gravity), or if the file failed to process (25, 26). Additionally, we excluded participants who had been diagnosed with dementia or PD before the end of their accelerometer wear.
Replication	This is a population-based cohort study, and we have not yet replicated the findings in other samples. However, we used different statistica methods to verify our findings: 1) controlling for a wide range of potential covariates; 2) comprehensive sensitivity analyses; 3) Stratified Analysis. Our main findings were quite robust and consistent across these analyses.
Randomization	This is an observational cohort study, and randomization is not applicable in this study.
Blinding	This is an observational cohort study, and randomization is not applicable in this study.

Reporting for specific materials, systems and methods

We require information from authors about some types of materials, experimental systems and methods used in many studies. Here, indicate whether each material, system or method listed is relevant to your study. If you are not sure if a list item applies to your research, read the appropriate section before selecting a response.

Materials & experimental systems

n/a	Involved in the study
☒	☐ Antibodies
☒	☐ Eukaryotic cell lines
☒	☐ Palaeontology and archaeology
☒	☐ Animals and other organisms
☒	☐ Clinical data
☒	☐ Dual use research of concern
☒	☐ Plants

Methods

n/a	Involved in the study
☒	☐ ChIP-seq
☒	☐ Flow cytometry
☒	☐ MRI-based neuroimaging
