# Supplementary material for: Association of accelerometer-measured physical activity intensity, sedentary time, and exercise time with incident Parkinson’s disease
Source: NPJ Digit Med. 2023 Nov 28;6:224. doi: 10.1038/s41746-023-00969-7 (PMC10684568; doi:10.1038/s41746-023-00969-7)
Supplement: Supplementary file 2 — Supplementary Information [file 41746_2023_969_MOESM2_ESM.pdf]

List of supplementary materials

Supplementary Figure 1. Flow chart of the participants in the current analysis.

Supplementary Figure 2. The dose-response associations of physical activity and sedentary behaviors with the risk of incident Parkinson's disease.

Supplementary Table 1. Sensitivity analysis for the independent associations of physical activity and sedentary time with the risk of incident Parkinson's disease.

Supplementary Table 2. The independent associations of physical activity and sedentary time with the risk of incident Parkinson's disease.

Supplementary Table 3. HR (95%CI) for incident Parkinson's disease associated with physical activity and sedentary time in various subgroups.

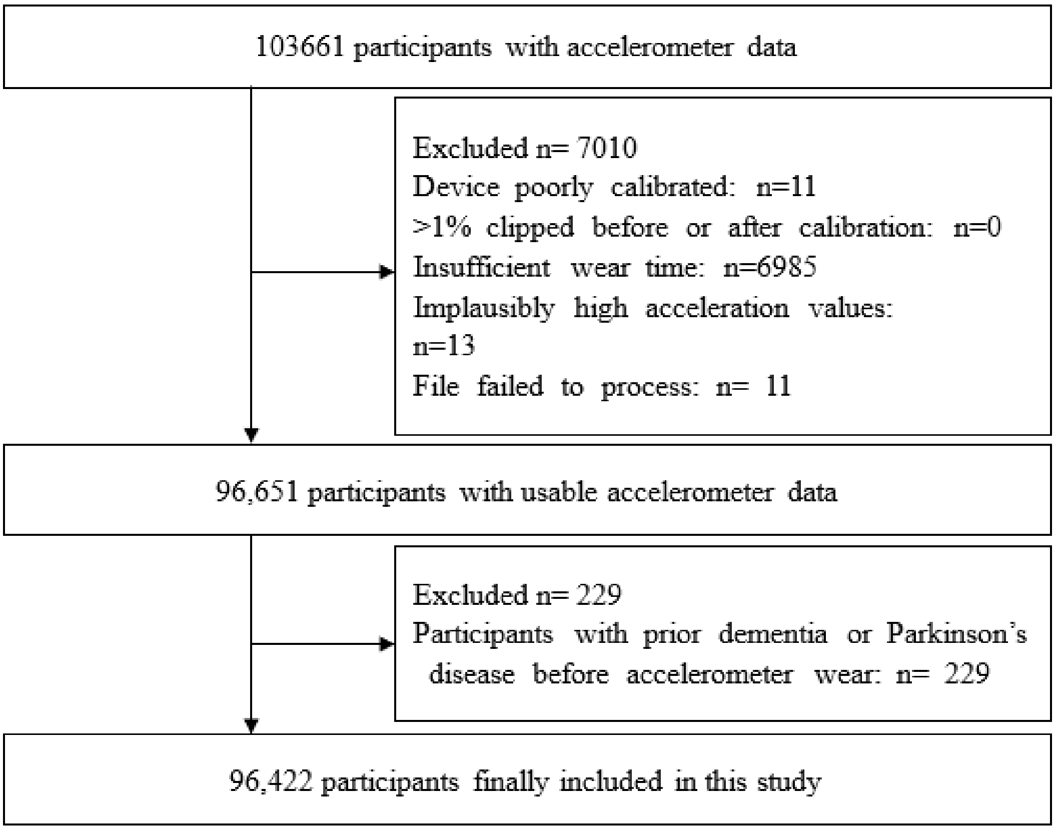

Supplementary Figure 1. Flow chart of the participants in the current analysis.

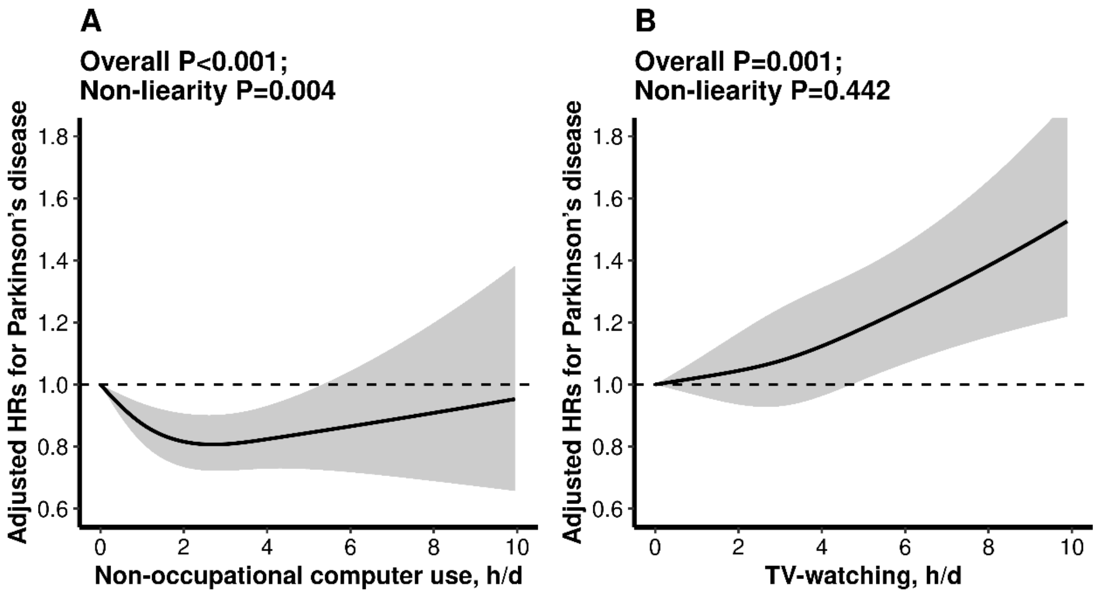

Supplementary Figure 2. The dose-response associations of sedentary behaviors with the risk of incident Parkinson's disease [1].

[1]Adjusted for age, sex, ethnicities, recruitment center, Townsend Deprivation Index, educational attainment, household income, employment, smoking status, alcohol consumption, and body mass index, and pre-existing chronic conditions (hypertension, diabetes and cardiovascular disease).

Supplementary Table 1. Sensitivity analysis for the independent associations of physical activity and sedentary time with the risk of incident Parkinson's disease.

	Quartiles of Exposures				*P* for trend
	**Q1**	**Q2**	**Q3**	**Q4**	
**Sensitivity analysis 1: Excluding participants within 2 years of follow-up**					
**Light physical activity**					
Events	153	49	33	25(1.6)	
Adjusted HR (95% CI)[1]	ref	0.33(0.23, 0.45)	0.22(0.15, 0.33)	0.19(0.12, 0.29)	<0.001
**Moderate-to-vigorous physical activity**					
Events	95	62	59	44	
Adjusted HR (95% CI) [1]	ref	0.60(0.43, 0.83)	0.55(0.39, 0.77)	0.37(0.26, 0.55)	<0.001
**Sedentary time**					
Events	36	43	64	117	
Adjusted HR (95% CI) [1]	ref	1.08(0.69, 1.68)	1.62(1.07, 2.45)	2.99(2.03, 4.41)	<0.001
**Sensitivity analysis 2: Using Fine-Gray competing risk model.**					
**Light physical activity**					
Events	193	55	35	30	
Death	1094	763	625	545	
Adjusted HR (95% CI) [1]	ref	0.29(0.22,0.4)	0.19(0.13,0.28)	0.18(0.12,0.27)	<0.001
**Moderate-to-vigorous physical activity**					
Events	119	72	66	56	
Death	1138	738	638	513	
Adjusted HR (95% CI) [1]	ref	0.57(0.43,0.77)	0.51(0.37,0.69)	0.39(0.28,0.55)	<0.001
**Sedentary time**					
Events	46	45	74	148	
Death	580	653	734	1060	
Adjusted HR (95% CI)[1]	ref	0.88(0.58,1.33)	1.45(0.99,2.11)	2.86(1.99,4.1)	<0.001

[1]Adjusted for age, sex, ethnicities, recruitment center, Townsend Deprivation Index, educational attainment, household income, employment, smoking status, alcohol consumption, and body mass index, pre-existing chronic conditions (hypertension, diabetes and cardiovascular disease), and the season of accelerometer wear.

Supplementary Table 2. The independent associations of physical activity and sedentary time with the risk of incident Parkinson’s disease.

Exposure	Total	Events (rate[1])	Crude Model		Adjusted Model[2]	
			HR (95%CI)	P value	HR (95%CI)	P value
**Light physical activity**						
Low (Q1)	24097	193(12.2)	4.89(3.89, 6.14)	<0.001	4.48(3.52, 5.69)	<0.001
High (Q2-Q4)	72325	120(2.5)	ref		ref	
**Moderate-to-vigorous physical activity**						
Low (Q1)	24089	119(7.6)	1.89(1.51, 2.38)	<0.001	2.07(1.62, 2.65)	<0.001
High (Q2-Q4)	72333	194(4.0)	ref		ref	
**Sedentary time**						
Low (Q1-Q2)	48195	91(2.8)	ref		ref	
High (Q3-Q4)	48227	222(7.0)	2.46(1.93, 3.14)	<0.001	2.28(1.77, 2.92)	<0.001

[1]Incidence rates per 10000 person-years.

[2]Adjusted for age, sex, ethnicities, recruitment center, Townsend Deprivation Index, educational attainment, household income, employment, smoking status, alcohol consumption, and body mass index, pre-existing chronic conditions (hypertension, diabetes and cardiovascular disease), and the season of accelerometer wear.

Supplementary Table 3. HR (95%CI) for incident Parkinson's disease associated with physical activity and sedentary time in various subgroups[1].

	Quartiles of Exposures				*P* for interaction
	Q1	Q2	Q3	Q4	
Light physical activity					
**Age, years**					0.373
<60	ref	0.35(0.18,0.70)	0.34(0.17,0.69)	0.22(0.10,0.50)	
≥60	ref	0.28(0.20,0.39)	0.16(0.10,0.24)	0.16(0.10,0.26)	
**Sex**					0.304
Female	ref	0.22(0.13,0.38)	0.14(0.08,0.25)	0.13(0.07,0.23)	
Male	ref	0.33(0.23,0.47)	0.22(0.14,0.35)	0.23(0.14,0.40)	
**Body mass index, kg/m^2**					0.730
<25	ref	0.35(0.22,0.57)	0.18(0.10,0.32)	0.20(0.11,0.35)	
≥25	ref	0.26(0.17,0.39)	0.21(0.13,0.33)	0.18(0.10,0.31)	
**Smoking status**					0.934
Never	ref	0.29(0.20,0.44)	0.19(0.12,0.31)	0.16(0.10,0.27)	
Ever	ref	0.29(0.18,0.46)	0.19(0.11,0.33)	0.21(0.11,0.38)	
**Alcohol consumption, times/week**					0.903
<1	ref	0.25(0.13,0.46)	0.18(0.09,0.37)	0.19(0.10,0.38)	
≥1	ref	0.31(0.22,0.43)	0.19(0.12,0.29)	0.17(0.11,0.28)	
**Townsend Deprivation Index**					0.673
< Median	ref	0.26(0.17,0.39)	0.17(0.11,0.28)	0.15(0.09,0.26)	
≥ Median	ref	0.34(0.22,0.53)	0.21(0.12,0.36)	0.22(0.12,0.38)	
Moderate-to-vigorous physical activity					
**Age, years**					0.868
<60	ref	0.49(0.25,0.97)	0.38(0.19,0.76)	0.28(0.14,0.58)	
≥60	ref	0.55(0.40,0.77)	0.50(0.35,0.70)	0.38(0.26,0.55)	
**Sex**					0.258
Female	ref	0.48(0.29,0.79)	0.30(0.16,0.56)	0.39(0.20,0.75)	
Male	ref	0.62(0.43,0.90)	0.60(0.41,0.86)	0.40(0.27,0.59)	
**Body mass index, kg/m^2**					0.365
<25	ref	0.49(0.30,0.82)	0.35(0.20,0.59)	0.33(0.20,0.55)	
≥25	ref	0.62(0.43,0.89)	0.62(0.43,0.91)	0.44(0.28,0.68)	
**Smoking status**					0.828
Never	ref	0.58(0.39,0.85)	0.48(0.32,0.73)	0.35(0.22,0.54)	
Ever	ref	0.55(0.35,0.87)	0.53(0.33,0.84)	0.45(0.28,0.74)	
**Alcohol consumption, times/week**					0.188
<1	ref	0.31(0.17,0.59)	0.43(0.24,0.78)	0.35(0.18,0.68)	
≥1	ref	0.68(0.48,0.95)	0.53(0.37,0.76)	0.40(0.27,0.59)	

**Townsend Deprivation Index**					0.936
< Median	ref	0.54(0.36,0.81)	0.46(0.30,0.70)	0.39(0.25,0.60)	
≥ Median	ref	0.58(0.38,0.90)	0.53(0.34,0.83)	0.37(0.22,0.61)	
Sedentary time					
**Age, years**					0.790
<60	ref	0.87(0.36,2.11)	1.08(0.48,2.46)	2.39(1.19,4.82)	
≥60	ref	0.93(0.58,1.48)	1.63(1.07,2.47)	3.22(2.18,4.74)	
**Sex**					0.178
Female	ref	0.94(0.47,1.89)	1.80(0.97,3.36)	4.56(2.58,8.06)	
Male	ref	0.82(0.49,1.37)	1.24(0.78,1.97)	2.28(1.51,3.46)	
**Body mass index, kg/m^2**					0.099
<25	ref	0.83(0.42,1.63)	1.62(0.90,2.94)	4.22(2.47,7.19)	
≥25	ref	0.83(0.49,1.40)	1.22(0.76,1.96)	2.12(1.38,3.27)	
**Smoking status**					0.786
Never	ref	0.83(0.49,1.40)	1.28(0.79,2.08)	2.86(1.85,4.41)	
Ever	ref	0.97(0.50,1.87)	1.72(0.96,3.07)	2.92(1.70,5.03)	
**Alcohol consumption, times/week**					0.189
<1	ref	1.56(0.73,3.30)	1.45(0.67,3.14)	3.35(1.70,6.60)	
≥1	ref	0.68(0.41,1.13)	1.44(0.94,2.19)	2.76(1.87,4.08)	
**Townsend Deprivation Index**					0.309
< Median	ref	0.62(0.35,1.10)	1.34(0.83,2.16)	2.63(1.70,4.07)	
≥ Median	ref	1.33(0.72,2.46)	1.64(0.91,2.96)	3.35(1.96,5.74)	

[1]Adjusted, if not stratified, age, sex, ethnicities, recruitment center, Townsend Deprivation Index, educational attainment, household income, employment, smoking status, alcohol consumption, and body mass index, pre-existing chronic conditions (hypertension, diabetes and cardiovascular disease), and the season of accelerometer wear.
